# Spontaneous splenic rupture in *Plasmodium knowlesi* malaria

**DOI:** 10.1186/s12936-018-2600-2

**Published:** 2018-12-04

**Authors:** Chee Yik Chang, Wei Chieng Pui, Khamisah Abdul Kadir, Balbir Singh

**Affiliations:** 1 Medical Department, Kapit Hospital, Ministry of Health, Kapit, Malaysia; 2 Surgical Department, Kapit Hospital, Ministry of Health, Kapit, Malaysia; 3 Malaria Research Centre, Faculty of Medicine and Health Sciences, Universiti Malaysia Sarawak, Kota Samarahan, Sarawak Malaysia

**Keywords:** Malaria, *Plasmodium knowlesi*, Splenic rupture, Splenectomy

## Abstract

**Background:**

*Plasmodium knowlesi,* a malaria parasite typically found in long-tailed and pig-tailed macaques, is the most common cause of human malaria in Malaysian Borneo. Infections in humans result in a spectrum of disease, including fatal outcomes. Spontaneous splenic rupture is a rare, but severe complication of malaria and has not been reported previously for knowlesi malaria.

**Case presentation:**

A 46-year-old man presented with fever and acute surgical abdomen with concomitant *P. knowlesi* malaria infection at Kapit Hospital. He was in compensated shock upon arrival to the hospital. He had generalized abdominal tenderness, maximal at the epigastric region. Bedside focused abdominal ultrasonography revealed free fluid in the abdomen. He underwent emergency exploratory laparotomy in view of haemodynamic instability and worsening peritonism. Intraoperatively, haemoperitoneum and bleeding from the spleen was noted. Splenectomy was performed. Histopathological examination findings were suggestive of splenic rupture and presence of malarial pigment. Analysis of his blood sample by nested PCR assays confirmed *P. knowlesi* infection. The patient completed a course of anti-malarial treatment and recovered well post-operation.

**Conclusions:**

Spontaneous splenic rupture is a rare complication of malaria. This is the first reported case of splenic rupture in *P. knowlesi* malaria infection. Detection of such a complication requires high index of clinical suspicion and is extremely challenging in hospitals with limited resources.

## Background

Malaria in humans was thought to be caused by four species of *Plasmodium* (namely *Plasmodium falciparum, Plasmodium vivax, Plasmodium ovale* and *Plasmodium malariae*) until a large focus of human infections with *Plasmodium knowlesi* was reported in 2004 in the Kapit Division of Sarawak, Malaysian Borneo [1]. *Plasmodium knowlesi* infections in humans have subsequently been reported throughout Southeast Asia and result in a spectrum of disease from very mild to fatal outcomes [2]. Malaria has been associated with various complications including liver or renal impairment, cerebral malaria and acute respiratory distress syndrome. Spontaneous rupture of spleen is a rare complication of malaria, occurring in only 0–2% of patients [3]. Most of the cases of spontaneous splenic rupture in malaria are associated with *P. vivax* although there have been rare cases associated with other *Plasmodium* species [4]. Of the 22 malaria cases with spontaneous splenic rupture reported in the literature since 1960, *P. vivax* was the most common (15 patients), followed by *P. falciparum* (5 patients) and *P. malariae* (2 patients) [5]. There has been no reported case of spontaneous splenic rupture due to *P. knowlesi.*

## Case presentation

A 46-year-old man, previously well, presented at Kapit Hospital, Sarawak, Malaysian Borneo with fever, chills and rigors for 2 days. It was associated with severe epigastric and left hypochondrium pain and loose stool. There was no preceding history of trauma. Upon arrival to the emergency unit, his general condition was stable. Physical examination revealed blood pressure of 123/86 mmHg, pulse rate of 114 beats/min and temperature of 39 °C. His respiratory rate was 23 breath/min and the oxygen saturation on room air measured by pulse oximetry was 97%. The abdomen was generally tender and guarded, maximal at the epigastric region. Bedside focused abdominal ultrasonography revealed free fluid in the abdomen. Chest radiograph did not reveal any obvious sign of pneumoperitoneum.

Haematological analysis showed haemoglobin of 11.5 g/dL, white blood cell count of 8.2 × 10^3^/μL and platelet count of 77 × 10^3^/μL. His creatinine level was 89 μmol/L and electrolytes were within the normal range. The results of the liver function tests were as follows: aspartate aminotransferase 15 U/L, alanine aminotransferase 11.8 U/L and total bilirubin 22.9 μmol/L. Serum amylase was normal. The arterial blood gas revealed good oxygenation and absence of metabolic disturbance (pH 7.44, PaO_2_ 87 mmHg, PaCO_2_ 34 mmHg, bicarbonate of 22.3 mmol/L and base excess − 1.8 mmol/L). The serum lactate measured was 0.8 mmol/L.

*Plasmodium knowlesi* was identified by examination of a Giemsa-stained blood film and the parasitaemia was estimated to be 240 parasites/μL blood. Finger prick blood samples from the patient were spotted on filter paper and sent to the Malaria Research Centre at Universiti Malaysia Sarawak where DNA was extracted as described previously [6]. The DNA was examined by nested PCR assays specific for *P. knowlesi*, *P. falciparum, P. vivax, P. malariae* and *P. ovale*, which indicated the patient was infected with *P. knowlesi* [7].

There was no formal radiological imaging done, such as abdominal ultrasonography or computed tomography, since this service is unavailable at Kapit hospital. Four tablets of artemether–lumefantrine were given upon laboratory diagnosis of *P. knowlesi* malaria.

The patient later developed hypotension despite fluid resuscitation and required single inotropic support, noradrenaline at a dose of 0.2 mcg/kg/min. He simultaneously experienced worsening peritonism. The surgical team was consulted, and he was posted for emergency exploratory laparotomy after stabilization. The anti-malarial was changed to intravenous artesunate in view that the patient was vomiting.

**Fig. 1:**
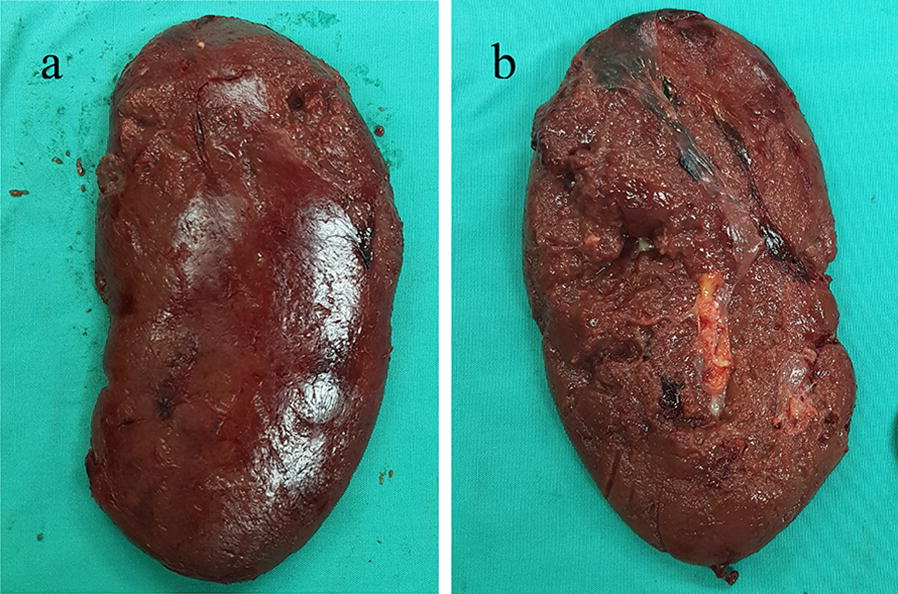
Gross appearance of the spleen. The diaphragmatic surface **a** shows tear of splenic capsule at the superior pole and the visceral surface, **b** shows subcapsular haematoma at the superior pole

Intraoperatively, haemoperitoneum was observed upon entering the abdomen, with blood clots seen overlying the splenic region. Gross examination of the spleen revealed tear of splenic capsule and subcapsular haematoma at the superior pole (Fig. 1). Otherwise, the spleen was of average size, measuring 12 cm. Total splenectomy was performed and no visceral perforation was seen. The estimated blood loss was 7 L. During the operation, he required high dose of inotrope infusion: noradrenaline up to 2 mcg/kg/min, adrenaline up to 1 mcg/kg/min and dobutamine constant at 5 mcg/kg/min, fluid resuscitation (2 L of crystalloid and 1.5 L of colloid solution) and disseminated intravascular coagulation (DIC) regime transfusion due to massive blood loss. Postoperatively, the patient was managed in the high dependency ward of Kapit Hospital. There, he remained stable, inotrope was weaned off and he was extubated on the next day after the operation. He was administered with intravenous artesunate for 4 days. It was later switched to oral artemether–lumefantrine which he completed following a total of 6 doses. Subsequent blood films were negative for malaria parasites.

Histopathological examination revealed splenic tissue with focal breaching of the capsule with areas of haemorrhage, consistent with splenic haemorrhage. There were a few brownish pigments within the spleen indicative of malarial pigment. The patient recovered well after the operation and was discharged after 9 days of hospital stay. He was given routine follow up post-malaria treatment and later administered with the routine post-splenectomy vaccinations against *Streptococcus pneumoniae*, *Neisseria meningitidis* and *Haemophilus influenzae*. One month later, he was well upon review at the surgical outpatient clinic.

## Discussion

Malaria can present with numerous clinical symptoms and complications. *P. falciparum* is recognized as the most common cause of severe complications in malaria [8]. However, many cases of severe malaria have been reported in *P. knowlesi* with approximately 1 in 10 patients developing severe complications, including fatal outcomes, at a district hospital in Sarawak, Malaysian Borneo [2].

Due to the relatively high prevalence of malaria in the Kapit division of Sarawak, there is a high suspicion for malaria in patients who came with febrile illness. In the present case, early diagnosis of *P. knowlesi* malaria was made in less than 2 h from presentation. After thorough assessment, suspicion of splenic rupture was raised based on clinical grounds and prompt operative intervention was performed. Therefore, early diagnosis of malaria, recognition of splenic rupture and prompt intervention have contributed to the favourable outcome in this patient.

Presentation of acute abdominal pain in malaria patients poses a great diagnostic challenge, especially in hospitals with limited resources. Left hypochondrium pain and circulatory shock are the commonest presentation of splenic rupture [9]. Imaging modalities such as ultrasound and CT serve as important tools in the detection of splenic complication. An abdominal ultrasound can detect subcapsular haematomas, perisplenic collections, splenic rupture and haemoperitoneum, whereas an abdominal CT scan is more accurate and more useful in diagnosis and monitoring [4]. Despite the absence of advanced diagnostic equipment, we have proven that malaria management in a peripheral healthcare setting is achievable by accurate and quick confirmation of malaria by microscopy and wise clinical observation and response by healthcare team comprised of physician, surgeon, anaesthetist, pathologist and laboratory staff.

Hyperparasitaemia is one of the criteria of severe malaria and the risk of severe knowlesi malaria increases 11-fold with parasitaemia > 20,000/μL and 28-fold with parasitaemia > 100,000/μL [10]. *Plasmodium knowlesi* parasitaemia upon hospital admission has also been shown to be an independent determinant of respiratory distress, renal and liver impairment [11]. However, this case has demonstrated that severe complications such as splenic rupture can occur in knowlesi malaria patients with low parasitaemia, just as reported for other low parasitaemia vivax malaria patients [12–14].

Traditionally, spontaneous splenic rupture due to malaria is treated with splenectomy [4]. However, recent advances in treatment and understanding of post-operative risk of splenectomy showed that conservative treatment can be applied in the setting of stable haemodynamics and lack of progression of haemorrhage [15, 16]. The current patient underwent splenectomy in view of massive bleeding from ruptured spleen leading to haemodynamic instability.

## Conclusion

Malaria remains as a major health problem worldwide. Spontaneous rupture of malarial spleen is rare. Nevertheless, failure to recognize and manage this complication early can adversely affect the patient’s outcome. Malaria management in poorly accessible healthcare services is extremely challenging and greatly dependent on clinical competence and experience.

## Abbreviations

CT: computed tomography
DIC: disseminated intravascular coagulation
